# Education intervention with respect to the oral health knowledge, attitude, and behaviors of refugee families: A randomized clinical trial of effectiveness

**DOI:** 10.1111/jphd.12415

**Published:** 2020-10-20

**Authors:** Murad Alrashdi, Ahmed Hameed, Maria Jose Cervantes Mendez, Moshtagh Farokhi

**Affiliations:** ^1^ Department of Orthodontic and Pediatric Dentistry, College of Dentistry Qassim University Buraydah Saudi Arabia; ^2^ Biology Department University of Texas San Antonio TX USA; ^3^ Department of Developmental Dentistry School of Dentistry at the University of Texas Health Science Center at San Antonio San Antonio TX USA; ^4^ Department of Comprehensive Dentistry School of Dentistry at the University of Texas Health Science Center at San Antonio San Antonio TX USA

**Keywords:** refugees, oral health, education, knowledge, attitude, behavior

## Abstract

**Objectives:**

The study assessed the effectiveness of an oral health educational and behavioral intervention program in improving the knowledge, attitudes, and behaviors of refugee families.

**Methods:**

This randomized 2‐arms, controlled, single site, clinical trial assessed the dental knowledge, attitudes, and behaviors related to oral health at baseline and three times over the course of the 6 months of the intervention in recent refugee families. Participating families were educated on five topics in oral health in two 1‐hour sessions utilizing existing oral health education materials adapted to be linguistically and culturally appropriate for demonstration and instruction. Culturally competent techniques and motivational interviewing styles were also implemented during sessions. Pre/post surveys were used to assess changes to knowledge, attitudes, and behavior among refugee family participants.

**Results:**

Out of the 66 families enrolled in the program, 52 (72 percent) completed visits over the course of 6 months. Differences between the intervention and control groups were not significant between baseline and 3 to 6 months later (*P* > 0.05).

**Conclusions:**

A short‐term, culturally informed oral health educational and behavioral intervention program did not improve oral health‐related knowledge, attitudes, or behaviors in a diverse group of recent refugee families.

## Introduction

Until only recently, the United States (U.S.) has led the world in the number of refugee families it resettles annually.^1^ Among the many health care needs of these refugee families, oral health issues rank near the top. In some cases, oral abnormalities were determined to be the most common health problem in refugee children resettled in the United States.^2^ Unfortunately, oral health issues among refugees in the United States are largely ignored, and in many cases go untreated, incurring greater risks of developing oral diseases among refugees.^3^

Poor oral health has a significant impact on quality of life and overall health outcomes.^4^ Untreated oral conditions like dental caries can interfere with everyday activities such as talking, eating, and sleeping.^5^ This is especially hard on refugee children as it can hinder academic performance and cognitive development.^6^ Several unfortunate circumstances make refugee children highly susceptible to having poor oral health. Such circumstances include low socioeconomic status, cultural and linguistic factors, poor diet and oral hygiene practices, lack of access to preventive dental services, and an absence of basic oral health education.^2^ Since newcomer populations are more likely to be uninsured, migrant and refugee parents generally consider dental examinations to be an additional financial burden.^3^ Since primary medical care is often prioritized over dental care, dental treatments are either delayed or ignored.^7^ Even when dental care is made available, refugee families tend to visit a dental office only if it is near their home due to transportation limitations.^2^ Consequently, refugee children tend to lack access to preventive oral health care materials such as toothbrushes or fluoridated toothpaste. As a result, refugee families are deficient when it comes to basic oral health knowledge about the positive effects of fluoride on oral health or the link between dental caries and sugary foods and drinks.^7^

To help reduce inequities in oral health care, educational programs have been touted as helping to promote good oral health practices in underserved communities.^6^ According to one review, some studies suggest that oral health education programs help enhance oral hygiene practices by improving oral health knowledge, attitudes, and behaviors.^8^ One study assessing the effectiveness of an oral health education program in improving such cognitive measures among migrants and underserved populations determined that educational programs were effective in enhancing oral health knowledge.^4^ Another study concluded that oral health education programs improved oral hygiene knowledge and behaviors in underserved Spanish‐speaking families, although they also revealed that a more rigorous assessment of the intervention was needed.^5^ Furthermore, a comprehensive evaluation of oral‐health promotional programs demonstrated an overall reduction in child dental caries. By increasing children's knowledge of favorable oral health behaviors, the programs helped decrease the cost of dental treatments on healthcare organizations, suggesting that they were not only clinically effective but also cost‐effective.^9^

Nonetheless, none of these studies specified if the oral health education programs were clinically effective or implemented among refugee populations. This is important since the oral health education of refugee parents and children specifically requires a culturally competent understanding of the oral health knowledge, attitudes, and behaviors of refugee communities.^10^ A recent review noted that, of the research studies that explored the oral health knowledge, attitudes, and behaviors of refugee families, only a small minority of studies discussed interventions to help improve such cognitive measures.^11^ For example, a study in Europe found that attitudes to and knowledge of preventive oral hygiene practices among refugees had improved as a result of an oral health education program.^12^ Nevertheless, the review suggested that more research is needed to assess the interventions in oral health education programs among different refugee populations.^11^

Overall, we found that there is limited research aimed at assessing an oral health education program's effectiveness in improving oral health cognitive measures among refugee families, especially in the United States. To address this issue, this study hypothesized that the administration of an oral health educational and behavioral intervention program that includes the demonstration and instruction of existing oral health education materials, culturally competent techniques, and motivational interviewing styles would improve oral health knowledge, attitudes, and behaviors of parents and children of refugee families. Therefore, this study sought to assess the effectiveness of an oral health educational and behavioral intervention program in improving oral health knowledge, attitudes, and behaviors among refugee families. The goal of this program is to both understand and assess methods to improve oral health cognitive measures among refugee participants using pre/post surveys to document oral health perspectives.

## Methods

### Study population

The study's target population consisted of children and caregivers of refugee families in Bexar County, San Antonio, Texas. Parents and caregivers from families that had at least one child under the age of 12 were recruited through the Center of Refugees' services in San Antonio. The study population spoke one of the following primary languages: Turkish, Burmese, Arabic, Nepalese, Spanish, and English. Although the majority of immigrants in the San Antonio area are Hispanic, we targeted recent refugees from a more geographically and culturally diverse group of countries, including Myanmar, Nepal, Turkey, Iraq, Afghanistan, Cameroon, and Eretria.

To detect a 20 percent difference between the control and intervention groups with a power of 80 percent and a significance level set to 0.05, a total of 55 participants was needed. To account for potential losses during the study, we recruited more than 60 participants for all measures of the study. This study was approved by the Institutional Review Board of University of Texas Health San Antonio (HSC20170703N). Written informed consent obtained from all participants of the study after randomization.

### Inclusion and exclusion criteria

Participants were included if recent refugees' caregivers had at least one child under age 12, were part of a refugee family and not staying with any other families, were residing in San Antonio, and did not plan to move away during 6 months study period. Participants were excluded if they were from refugee families that had spent more than 1 year in the United States and had already established dental visits.

### Study procedures

The baseline survey that focused on one caregiver and one child per family was performed for all participants upon enrollment at Time 1 (T1) of the project and immediately after the education session for the intervention group only at Time 2 (T2). Two follow‐up visits to assess the impact of the intervention were performed at 3 months at Time 3 (T3) and 6 months at Time 4 (T4) for the intervention group, and at T4 only for control group. All surveys were interviewer‐administered by trained, multilingual research assistants in one of the six primary languages mentioned above.

Upon enrollment, study participants were randomly divided into two groups. Those in the intervention group agreed to attend five education sessions and four evaluations. The control group consented to complete two study evaluations without an intervention. With regard to the allocation of the participants into groups, a computer‐generated list of random numbers was used. The balance between the two arms was assessed by comparing demographic and outcome measures at baseline using two‐sample t‐tests. Participants and educators were aware of each child's allocation, but outcome assessors and data analysts were kept blinded to the allocation.

### Educational and behavioral intervention

Since oral health education of refugee parents and children is better achieved through a culturally competent understanding of the oral health knowledge, attitudes, and behaviors of refugee communities, cultural competency techniques were used for the educational program and intervention. An earlier review presented a model that can be implemented by health‐care organizations to use culturally competent techniques in their programs.^13^ These techniques include recruitment and retention policies for staff members that reflect the cultural diversity of the community being served, use of interpreter services and bilingual providers for non‐English speaking patients, cultural competency training for healthcare providers and assistants, and use of culturally and linguistically appropriate oral health education materials.^13^, ^14^, ^15^ These techniques were used at various stages of the intervention.

A culturally focused educational program was administered following enrollment using two guides. The first guide, “A Healthy Mouth for Your Baby,” was developed as an educational tool by the National Institute of Dental and Craniofacial Research, U.S. Department of Health and Human Services (DHHS), and consists of a brochure for parents and other caregivers discussing oral health in young children; the topics include the importance of primary teeth and the role of fluoride and oral hygiene in preventing tooth decay, tips on checking and cleaning teeth, feeding and nutrition, and the importance of having a dental visit by age 1.^16^ This material was reviewed by the NIH Nutrition Education Subcommittee (NES) and underwent a joint review by the DHHS Nutrition Policy Board Committee on Dietary Guidance and the U.S. Department of Agriculture (USDA) Dietary Guidance Working Group.^17^

The second guide is “Healthy Habits for Happy Smiles,” which consists of a series of handouts to promote good oral health in pregnant women and parents of infants and young children by providing simple tips on oral health issues. These resources were prepared by the National Center on Early Childhood Health and Wellness under the cooperative agreement #9OHC0013 between the U.S. Department of Health and Human Services, Administration for Children and Families, and Office of Head Start.^18^ All the educational material in this program was translated into the native languages of each refugee family. With consultation from interpreters and members of the refugee community, both of these guides were edited to be both culturally and linguistically appropriate for each of the refugee participants in the program.

This oral health education program included several techniques that aimed to improve oral health knowledge, attitude, and behavior. The main techniques we used were instruction, demonstration, and motivational interviewing. The oral health education program implemented two 1‐hour sessions of demonstrations and instructions using colored visuals emphasizing the following topics related to the practice of good oral health behaviors: the importance of fluoride, oral hygiene, nutrition, oral health, and access to dental care. Demonstrations of good oral health behaviors such as flossing, using mouthwash, and brushing teeth were performed to help participants of the intervention group visually understand how to perform them correctly. We also presented information about the consequences of inaction regarding good oral health behaviors. Such consequences included detailing what happens when someone does not take care of their oral health. The benefits of performing good oral health behaviors was also discussed. Demonstrations and instructions were carried out by research assistants and interpreters from diverse cultural backgrounds, reflecting the diversity of the refugee community being served. Emphasis was placed on conducting the interventions with the help of volunteer interpreters from the refugee community who have a better understanding of the refugee experience. Research assistants were trained for the educational program by dental professionals and participated in group discussions with interpreters and members of the refugee community to implement a way in which the educational materials could be delivered in a culturally and linguistically appropriate manner. In addition, motivational interviewing style was employed by research assistants and interpreters during trial sessions to help participants evaluate their own oral health behavior, resolve ambivalence, and self‐motivate changes to their oral health behavior. With limited evidence, a 2009 Review suggested that motivational interviewing may help improve oral health outcomes (Martins &McNeil, 2009). Participants in our trial intervention group were given the choice to implement good oral health behaviors or not. At the end of the session and each visit, we provided general encouragement by rewarding the refugee participants with toothbrushes, toothpaste, flosses, and educational brochures. However, this was done for both the intervention and control groups without any contingency for their oral health knowledge, attitudes, or behaviors. In accordance with the protocol, control participants received the oral health educational and behavioral intervention program at the end of the study.

### Outcome measurements

#### Assessment of caregivers' knowledge, attitudes, and behaviors

The questionnaires included a variety of oral health topics to assess the knowledge, attitudes and behaviors of caregivers' oral health. These questionnaires were previously developed by a 7‐year longitudinal study being conducted by the Detroit Dental Health Project (DDHP) and validated by external research.^19^, ^20^, ^21^, ^22^ In order to assess the knowledge, attitudes, and behaviors of oral health, we probed different questions aimed at assessing these variables.

#### Knowledge of oral hygiene

General oral health knowledge was measured with 10 items, covering the content of all five educational sessions. Correct answers were recoded as “1”, wrong answers or “I don't know” answers as “0,” and no existing knowledge was indicated by participants. A subscale sum score for oral hygiene‐behavior related knowledge was computed (10 items; min. 0, max. 10). This score reflects correct answers with regard to the promoted frequency and appropriateness of brushing and flossing, using fluoride toothpaste, cleaning teeth before sleeping, and rinsing with mouthwash. The oral health knowledge score was calculated such that one point was given for each correct answer and no point was given for an incorrect answer, do not know or refused to answer. The sum was then obtained. A higher knowledge score is desirable on a range of 0–10.

#### Attitude toward oral health

Nine statements were used to measure a range of oral health attitudes, with participants selecting “Disagree,” “Neutral,” or “Agree” answers. Each question referred to one attitude construct toward oral health, for example, perceived susceptibility or fatalistic belief. The attitude score was calculated such that, for each question, two points were given for demonstrating a positive attitude, one point for a neutral attitude, and no points for a negative attitude. We then took the sum across all questions. A higher attitude score is desirable on a range of 0–18.

#### Oral hygiene behavior

During the program, the proper use of toothbrush, toothpaste, and dental floss was promoted. Nine questions on a 4‐point Likert scale (1 =“Never,” 2 = “Rarely,” 3 = “Sometimes,” 4 = “Usually”) were used to capture the frequency of a range of hygiene behaviors. Participants were asked about the type of rinsing medium used to clean their mouths (using water, saltwater, and mouthwash), toothpaste use (using no toothpaste, fluoride toothpaste, non‐fluoride toothpaste, something else other than toothpaste), use of dental floss, and use of toothpicks. The behavior score was calculated such that, for each question, one point was given when the response was “Rarely,” two points for “Sometimes,” three points for “Usually,” and 0 points for all other options. We then computed the sum across all questions in this section. A higher behavior score is desirable on a range of 0–27.

### Statistical analysis

Statistical analyses were calculated using SPSS statistical package version 23 (SPSS Inc, Chicago, IL). The Generalized estimated equation (GEE) method was used to test the hypothesis that the educational program would have a positive influence on the oral health knowledge, attitudes, and behaviors of refugees families.^23^ The multivariate model (GEE) was used to analyze the longitudinal data. It estimates regression parameters that allow for the specification of a working correlation matrix to account for a within‐subjects correlation of dependent variables responses with many different distributions.

Each parameter was analyzed according to its nature to select the most suitable working correlation matrix. Model selection regarding the distribution of the residual error was performed for all outcomes and the distributions that yielded the lowest quasi‐likelihood information criterion (QIC) scores were chosen. An exchangeable correlation matrix was selected as the best type of matrix. Covariates of interest addressed in this study were socioeconomic status (monthly household income) and education in order to test the hypothesis that refugee families with a higher socioeconomic and education status would show better improvement. A Cronbach's alpha was used to evaluate the reliability of the survey after averaging and summarizing the knowledge, attitude, and behaviors scale.

## Results

### Participant demographics

The majority (74 percent) of parental/caregiver participants were women, most (96 percent) were married or living with a partner, 51 percent had completed high school, and the majority of participants (71 percent) had a monthly income under $2000 per month. Detailed participants' demographic characteristics at baseline are presented in Table 1.

**Table 1:** Demographic Characteristics of the Parents/Caregivers

Variables	Intervention group	Control group	Total
	% (n)	% (*n*)	% (*n*)
Gender			
Male	23 (7)	37 (13)	30 (20)
Female	77 (24)	63 (22)	60 (46)
Marital status			
Married/living with partner	94 (29)	100 (35)	97 (64)
Single/separated/divorced/widowed	6 (2)	0 (0)	3 (2)
Education			
Elementary or middle school	52 (16)	49 (17)	50 (33)
High school	19 (6)	17 (6)	18 (12)
Some college but not a degree	19 (6)	6 (2)	12 (8)
Bachelor's degree	7 (2)	17 (6)	12 (8)
Master's degree	3 (1)	11 (4)	8 (5)
Race			
Southeast and South Asian	58 (18)	60 (21)	59 (39)
Middle Eastern	32 (10)	20 (7)	26 (17)
African/Hispanic	10 (3)	20 (7)	15 (10)
Country of origin			
Myanmar	49 (15)	31 (11)	39 (26)
Nepal	26 (8)	14 (5)	20 (13)
Turkey	13 (4)	20 (7)	17 (11)
Iraq	3 (1)	20 (7)	12 (8)
Afghanistan	3 (1)	6 (2)	4 (3)
Cameroon	3 (1)	3 (1)	3 (2)
Eritrea	3 (1)	3 (1)	3 (2)
Mexico	0 (0)	3 (1)	2 (1)
Language			
Burmese	48 (15)	31.4 (11)	39 (26)
Nepalese	26 (8)	14.3 (5)	20 (13)
Turkish	13 (4)	20 (7)	17 (11)
Arabic	3 (1)	20 (7)	12 (8)
English	10 (3)	12 (4)	10 (7)
Spanish	0	3 (1)	2 (1)
Monthly income			
<$2000	64 (20)	80 (28)	73 (48)
>$2000	36 (11)	20 (7)	27 (18)
Age			
18–24 years	16 (5)	17 (6)	17 (11)
25–34 years	45 (14)	49 (17)	47 (31)
35–44 years	29 (9)	31 (11)	30 (20)
>45 years	10 (3)	3 (1)	6 (4)
Child's age			
3–5 years	29 (9)	28 (10)	29 (19)
6–8 years	45 (14)	43 (15)	44 (29)
9–11 years	23 (7)	26 (9)	24 (16)
12 years	3 (1)	3 (1)	3 (2)

**Figure 1:**
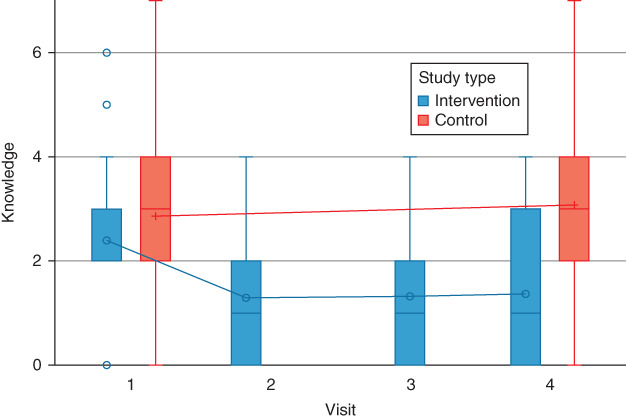
Flow diagram representing the recruitment of participants in the intervention and control groups. The study was a single‐center trial with a parallel, randomized group design. [Color figure can be viewed at wileyonlinelibrary.com]

At baseline, 66 participants were enrolled and completed the baseline survey (T1) of 75 families approached and assessed for eligibility, 31 were assigned to the intervention group, and 35 to the control one. The survey was repeated in the intervention group for 31 participants at T2 and 28 participants at T3, and for 52 participants at T4. There were 22 in the intervention group and 30 in the control group who completed the trial and included in the study's analysis. The dropout rate was 28 percent at the 6‐months (T4) follow‐up. Potential reasons for dropping out were tracked when possible and included losing contact with participants, poor cell phone reception and accessibility, migration to another city for work, and lack of transportation. The distribution of the study groups and sample size across the four measurement time points are displayed in Figure 1.

### Randomization verification and survey reliability

Two‐sample t‐tests were used to compare the outcomes before intervention at T1 between the intervention and control groups. All *P*‐values were > 0.6; therefore, we concluded that the randomization was successful. The Cronbach's alpha coefficient was calculated to assess knowledge, behavior, and attitude survey sections in order to measure internal consistency. The standardized Cronbach's alpha coefficient was 0.76 for knowledge, 0.74 for attitude, and 0.77 for behavior. These results demonstrate good internal consistency for the survey.

### Outcomes

**Figure 2:**
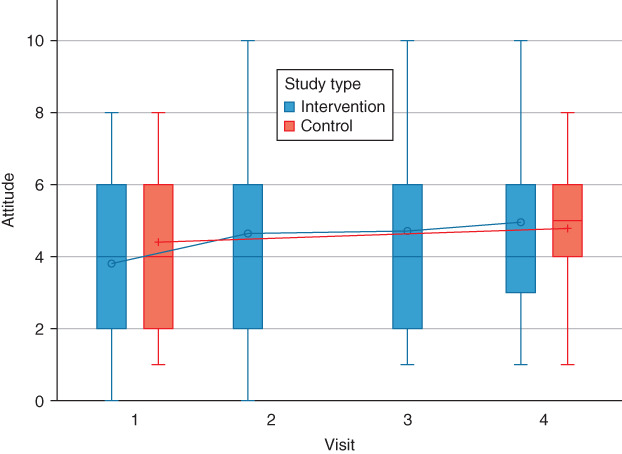
Distributions of knowledge scores at each time point in the intervention and control groups. [Color figure can be viewed at wileyonlinelibrary.com]

**Figure 3:**
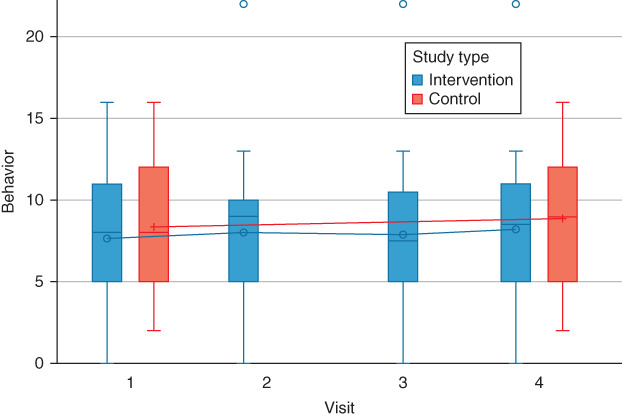
Distributions of attitude scores at each time point in the intervention and control groups. [Color figure can be viewed at wileyonlinelibrary.com]

**Figure 4:**
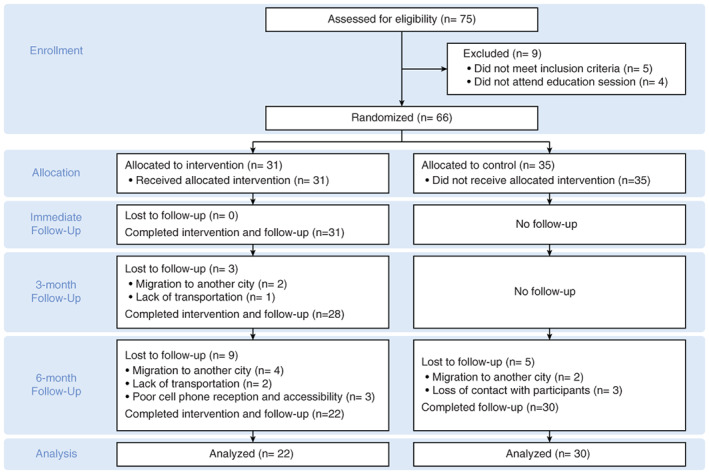
Distributions of behavior scores at each time point in the intervention and control groups. [Color figure can be viewed at wileyonlinelibrary.com]

The knowledge, attitude, and behaviors of parents pre‐ and post‐educational program did not show any improvements in either group. The regression coefficient estimate and 95% CI of the interventional program were *β* = 0.0202, 95% CI (−0.0049, 0.0452), *P* = 0.1142 for knowledge, *β* = 0.1392, 95% CI (−0.0016, 0.2801), *P* = 0.0527 for attitude, and *β* = 0.0132, 95% CI (−0.0888, 0.1153), *P* = 0.7992 for behavior. With respect to the covariates in the multivariate model, socioeconomic status was significantly associated with knowledge, and parents who had a higher income experienced a negative improvement in knowledge (*β* = −0.0627, 95% CI (−0.1135, −0.0119), *P* = 0.0156). Detailed multivariate analyses are shown in Table 2. The distribution of knowledge, attitudes, and behaviors at each time point in the intervention and control groups are shown in Figures 2, 3, 4.

**Table 2:** Multivariate Analyses for the Predictors Estimating Oral Health Knowledge, Attitudes, and Behaviors

Outcome	Parameter	Estimate	95% Confidence Limits		*P*‐value
Knowledge	Treatment	0.0428	−0.0074	0.0930	0.0946
	Time (T4 versus T1)	0.0202	−0.0049	0.0452	0.1142
	Income (high versus low)	−0.0627	−0.1135	−0.0119	**0.0156**
	Education	0.0104	−0.0043	0.0251	0.1649
Attitude	Treatment	−0.0491	−0.2750	0.1767	0.6697
	Time (T4 versus T1)	0.1392	−0.0016	0.2801	0.0527
	Income (high versus low)	−0.1519	−0.4129	0.1090	0.2539
	Education	0.0272	−0.0443	0.0988	0.4555
Behavior	Treatment	−0.0682	−0.3000	0.1637	0.5644
	Time (T4 versus T1)	0.0132	−0.0888	0.1153	0.7992
	Income (high versus low)	0.2523	−0.0891	0.5937	0.1475
	Education	−0.0704	−0.1450	0.0043	0.0646

## Discussion

This article presented the results of a randomized clinical trial assessing the effectiveness of an oral health educational and behavioral intervention program in improving the oral health knowledge, attitudes, and behaviors of refugee families. We did not find any clinically important or statistically significant difference in knowledge, attitude, or behavior among the intervention and control groups of refugee participants in this study. As the intervention was implemented for refugee parents living in the United States for 1 year or less with at least one child under the age of 12 and from countries such as Myanmar, Nepal, Turkey, Iraq, Afghanistan, Cameroon, and Eretria with no established dental visits, the results indicated that a pre‐ and post‐oral health educational and behavioral intervention program alone did not improve the oral health knowledge, attitudes, and behaviors of refugee families in our composite measures. We covered a broad spectrum of refugee families by including participants from a culturally and geographically diverse group of nations. Therefore, the trial findings suggest that the present oral health educational and behavioral intervention program implemented in this study is not effective when applied to refugee families living in the United States for 1 year or less with at least one child under the age of 12 and with no established dental visits. The conclusions of this trial are not applicable to oral health interventions delivered to other populations.

Although both the randomization and internal consistency of the survey were successful, the results of this study were not consistent with other studies that showed improvements in oral health knowledge, attitudes, and behaviors as a result of oral health education programs. However, some of those studies lacked a control group when conducting their trials. They also focused on the effectiveness of oral health education programs among migrants and immigrants in general, rather than specifically on refugees.^4^, ^5^

Studies that do show improvements among refugee families not only included oral health education programs as an intervention but also additional interventions such as providing dental screenings, treatments, sealants, and applications of topical fluoride varnishes.^9^ For instance, a study by Zimmerman et al. that showed an improvement in oral health knowledge and attitudes among refugees in Europe noted that oral prophylaxis treatments were used in addition to the oral health education programs.^12^ These additional interventions may have confounded the effects of the oral health education programs.^9^ Thus, the education programs alone were not shown in those studies to elicit the same results in improving the oral health knowledge, attitudes, and behaviors among refugee families. A comprehensive review of the effectiveness of oral health education programs determined that oral prophylaxis treatments, along with sustained oral health education programs, were usually more effective in improving oral health outcomes in long‐term studies.^8^ Additionally, one review recommended that oral health education programs should be supported by multiple other health promotion interventions since oral health education alone is of limited value.^8^

Furthermore, studies have linked a decline in refugee oral health to less access to preventive care and lower adherence to beneficial oral hygiene practices.^2^ Moderately acculturated refugees are at risk of adopting cariogenic Western dietary habits, making them more susceptible to poor oral health practices. It is especially deleterious to oral health when refugees do not also adopt the preventive aspects of Western oral hygiene and do not have adequate access to Western dental treatments.^2^ This is consistent with our findings that oral health education alone may not be effective at improving the oral health outcomes of refugees. Other interventions such as preventive, restorative, and curative dental treatments are needed in addition to educational programs to further improve the attitudes and behaviors toward oral health.^8^

Although we used common behavioral change techniques similar to those discussed in an article about oral health behavior interventions, our study was not successful in changing outcomes for oral health behavior.^23^ An important part of our oral health behavior interventions was motivational interviewing. However, one review noted that motivational interviewing had conflicting results for improving oral health behavior.^24^ Previous studies yielding positive results for motivational interviewing interventions were conducted in clinical settings and applied interventions in an individually tailored manner.^24^ In contrast, a study done with a greater community sample size did not yield positive results.^24^ The review concluded that motivational interviewing interventions were designed to improve behavioral change at the individual level in clinical settings and may not improve changes at the population level.^24^ The motivational interviewing technique in our trial was not individually tailored to each participant and was not performed in a clinical setting. The results of our study are in agreement with the conclusions of the review by^24^ since our population‐level behavioral intervention did not produce any significant positive results. In addition, our trial had low fidelity to the original motivational interviewing model based on the works of.^24^, ^25^ We did not have a fidelity assessment to make sure the interviewers were properly implementing the methods of motivational interviewing. Fidelity assessments for the protocol of the demonstration and instruction parts of the educational and behavioral interventions were also lacking. These deficiencies may also explain why our behavioral intervention was not successful. Additionally, a previous population‐level study involving behavioral interventions to prevent childhood caries cited the lack of dental treatments during the intervention and the poor accessibility of dental care in the community as some of the reasons for why their behavioral intervention was not effective.^26^ Our trial also exhibited a lack of dental treatments in addition to the educational and behavioral interventions.

Although no improvement in cognitive measures was observed among the intervention and control groups, an inverse relationship between socioeconomic status and knowledge was identified. As income level increased, improvements in oral health knowledge decreased. In fact, refugee parents who had a higher income level also experienced a negative improvement in knowledge. This was observed after the analysis for socioeconomic status was adjusted. These results were inconsistent with the results of other studies which showed that population groups with the worst oral health status were also those with the lowest income and education levels.^27^ Indeed, people of lower income and education levels face higher rates of untreated dental abnormalities and regularly self‐report poor oral health status.^28^ In general, higher incomes enable access to oral health services and a higher education offers the ability to better obtain and understand information regarding oral health promotion and behavior.^24^ Based on the results of previous studies, we assumed that refugee parents with higher income and education levels should have experience greater improvements in oral health outcomes in terms of their knowledge, attitudes, and behaviors.^24^ However, this was not apparent in our study. The data from our study suggested that they may improve their oral health knowledge to the same degree, or not quite as strongly, as refugee parents of lower income levels. In our view, it may be that refugees with higher incomes are preoccupied with job responsibilities and more focused on increasing their socioeconomic status than on retaining information from an oral health education program. However, this is only an assumption. These results may just reflect a practical problem in the measurement of incomes. Income can fluctuate over time. Therefore, income reported in a given time period may not accurately reflect the overall socioeconomic status of the individual in the long run.^24^ Other issues with the measurement of income are related to the high nonresponse rates regarding personal and household income, and the possibility that participants may not report real incomes.^24^

This study's findings need to also be interpreted in light of some limitations associated with the low power of the study in detecting small effects of the intervention since our small sample size was based on our choice of a large, targeted effect size. Another limitation was the possible cross‐contamination between the intervention and control groups. It was not possible to control due to the nature of the intervention and the strong social connection between participants since they resided in the same area. However, based on the study's findings, the chances of contamination were very slim since there were no positive responses to the educational program. Nonetheless, if there had been contamination, the effect of the control group would have had a greater influence on the intervention group.

Despite these limitations, this study demonstrated that an oral health education program was not effective in improving the oral health knowledge, attitudes, or behaviors of refugee families. We recommend multiple interventions, such as oral prophylaxis and other dental treatments, to be used in conjunction with a sustained oral health education program to achieve more favorable results in improving oral health knowledge, attitudes, and behaviors in refugee communities. According to our analysis, an oral health education program can be supplemental with other interventions but may not be sufficient by itself. Nevertheless, studies evaluating the effectiveness of oral health education programs with more extended follow‐up periods and higher power sizes for refugee families are needed.

## Conflict of interest

The authors have no conflict of interest to declare.
